# Association between Age, Sex and Cervical Spine Sagittal Plane Motion: A Descriptive and Correlational Study in Healthy Volunteers

**DOI:** 10.3390/life13020461

**Published:** 2023-02-07

**Authors:** Carlos Antonio Zárate-Tejero, Pere Ramón Rodríguez-Rubio, Lindsay Brandt, John Krauss, Mar Hernández-Secorún, Orosia Lucha-López, César Hidalgo-García

**Affiliations:** 1 Physiotherapy Department, Faculty of Medicine and Health Sciences, Universitat Internacional de Catalunya, 08195 Barcelona, Spain; 2 School of Health Sciences, Oakland University, Rochester, MI 48309, USA; 3 Unidad de Investigación en Fisioterapia, Universidad de Zaragoza, 50009 Zaragoza, Spain

**Keywords:** cervical spine, cervical flexion and extension, upper cervical flexion and extension

## Abstract

Active motion examination of patients with cervical spine-related pathologies is necessary to establish baseline function, set physical therapy goals, and choose interventions. This study investigated the sagittal plane active range of motion (ROM) of the global (GCS) and upper cervical spine (UCS) in relation to age and sex in healthy volunteers. One hundred twenty-two volunteers aged 18 to 75 years participated. Volunteers were excluded if they displayed any characteristic that could affect cervical spine ROM. GCS and UCS flexion and extension were each measured three times using a CROM device. Linear regression models (LRMs) were developed to explore the relationships between age and sex and GCS and UCS ROM. The LRM for age showed a decrease in GCS flexion (−2.01°), GCS extension (−3.33°), and UCS extension (−1.87°) for every decade of increasing age. The LRM for sex showed that men displayed less ROM than women in GCS extension (−6.52°) and UCS extension (−2.43°). These results suggest an age-related loss of sagittal plane GCS ROM and UCS extension ROM, and sex-related differences in GCS and UCS extension with women having greater motion than men.

## 1. Introduction

Effective clinical examination of patients with cervical spine-related pathologies is necessary to establish baseline function, guide physical therapy goals and intervention, and assess patient progress [1,2]. Components of this examination include measures of mobility, posture, strength, and stability, with comparison to reference values when available [1,3].

The cervical spine may be divided into two anatomical regions—the upper cervical spine and the lower cervical spine—each with unique characteristics. With respect to mobility, the primary function of the upper cervical region—formed anatomically by the occipital bone, atlas (C1 vertebrae), and axis (C2 vertebrae)—is to allow rotation, although movement in the sagittal plane has also been described [4,5]. The lower cervical spine is formed by the C3-C7 vertebrae and has primary functions of flexion and extension, while also allowing lateral flexion and rotation [6].

Several studies and systematic reviews have described the global cervical spine (GCS) active range of motion (AROM) in the three cardinal planes in healthy individuals and have proposed reference values [7,8,9,10]. Thoomes-de Graaf et al. conducted a systematic review of studies investigating cervical spine AROM in healthy participants using non-radiological means [9]. The review included 55 articles using 16 different measurement systems, the results of which cumulatively demonstrated greater AROM in the first and second decades of life with a progressive decrease until the sixth decade, though no statistical comparison was reported. Pan et al. conducted a systematic review and meta-analysis of studies investigating the effect of age and sex on cervical spine mobility [8]. The review included 34 studies that assessed cervical spine AROM in asymptomatic participants aged 20 to 70 years, of which 11 non-radiological studies were included in a meta-analysis. The meta-analyzed results suggest various differences in AROM related to age and/or sex and include the magnitude of difference between groups but do not report AROM reference values for each group. The meta-analysis indicates a heterogeneity of data that varied between 0% and 94%, with 11% having substantial heterogeneity [8]. Chen et al. analyzed the results of 37 studies that measured cervical spine AROM in asymptomatic subjects and compared the values obtained with each of nine different measurement systems which included visual estimates in addition to various types of radiographic and non-radiographic technologies. A narrative review of the influence of age and sex on cervical spine AROM suggests agreement across the studies that women have greater AROM than men and that AROM decreases with age at a rate of four degrees per decade, though no statistical calculations were reported [7].

Measurement of AROM isolated to the upper cervical spine (UCS) is also valuable in guiding physical therapy intervention for individuals with symptoms and/or pathology in the cervical region. Evaluation of ROM is a standard test and measure used during the examination of patients with cervical spine disorders [11,12] allowing the establishment of baseline function, the establishment of treatment objectives, and the evaluation of patient progression [1,2]. Thus, researchers have begun to investigate AROM in this anatomic sub-region. Dhimitri et al. reported UCS flexion AROM of 4° (+1.4) and extension AROM of 11.2° (+1.8) in 30 healthy participants aged 23–37 years using the Cervical Range of Motion (CROM) system [13]. Amiri et al. measured sagittal plane UCS AROM in 21 asymptomatic subjects aged 20–50 years using an electromagnetic device and reported flexion AROM of 15.03° (+2.80) and extension AROM of 23.32° (+4.20) [14].

Ernst et al. investigated sagittal plane UCS AROM in participants with neck pain and compared it with degree of disability using the Neck Disability Index (NDI) [15]. The study included 19 participants with a mean age of 29.2 (+10.3) years and a mean pain duration of 3 years. The authors reported a correlation between NDI score and UCS AROM that was higher than the correlation between NDI score and GCS AROM, suggesting UCS AROM is a relevant measurement in the assessment of patients with cervical region pain.

Existing literature presents wide variability in sagittal plane cervical spine AROM values when considering global movement as well as movement isolated to the UCS. This variability may be due to differing methodologies used to determine the measurement angle reported or differences in the success of preventing movement in the lower cervical spine during UCS AROM measurement [13]. No studies were located that investigated UCS AROM with consideration of age and sex in healthy, asymptomatic participants. Reference values for cervical spine AROM obtained using measurement procedures that are reproducible by physical therapists in clinical settings are necessary to accurately identify the existence of cervical spine motion limitations in patients seeking physical therapy for cervical spine complaints.

The purposes of this study were to describe sagittal plane GCS AROM and UCS AROM in adults without cervical pathology, stratified by age and sex, and to compare GCS AROM with UCS AROM. The hypotheses are as follows: (1) there is a relationship between UCS AROM and age and sex in persons without cervical pathology; (2) there is a relationship between GCS AROM and age and sex in persons without cervical pathology; and (3) there is a relationship between GCS AROM and UCS AROM in persons without cervical pathology.

## 2. Materials and Methods

Following ethics committee approval from the Universitat Internacional de Catalunya (UIC), 122 adults were recruited for this prospective cross-sectional observational study. Participants were recruited through an email disseminated to the faculty and students of the UIC Barcelona campus and paper advertisements on the same campus. Those interested in participating were directed to contact the primary investigator who performed a telephone interview to review the study and screen for eligibility. Men and women aged 18–75 years were eligible to participate. Individuals reporting or displaying one or more of the following criteria were excluded: history of headaches [16,17] more than once per month, cervical pain requiring treatment by a healthcare provider in the last year, history of surgery in the cervical region, known congenital alterations in the cervical spine, whiplash or traumatic brain injury in the last year, presence of chronic systemic diseases or acute infections at the time of the study, confirmed or suspected pregnancy, language limitations that impeded understanding the informed consent in Spanish, a positive result on any of the cervical spine stability tests, and inability to perform one or more of the evaluation techniques during the study.

Following confirmation of eligibility, participants attended a single data collection session at the UIC Barcelona physiotherapy functional assessment room. Upon arrival, the study procedures were described, all participant questions were answered, and informed consent was obtained.

### 2.1. Sample Size Calculation

Sample size was calculated with GRANMO-IMIM 7.12 online version using the averages menu for a population estimate, with a confidence level of 0.95 for a population of 47 million. It was calculated according to a standard deviation of 16° for the AROM variables [10] with an accuracy of three units for GCS AROM [18]. Results of these calculations suggested a total sample size of 110, and the choice was made to recruit 10 subjects per sex for each of six age groups between 18 and 75 years of age (18–25, 26–35, 36–45, 46–55, 56–65, 66–75), resulting in a total of 120 subjects sought for the study.

### 2.2. Materials and Procedures

Following informed consent, the participant was interviewed by the researcher to obtain and record the following information: age in years, sex, medication intake, presence of neck pain in the last year, presence of neck pain at the time of interview, daily hours of data display screen use, daily hours spent sitting, weekly hours of physical activity, existence of visual impairment, and use of a dental splint for bruxism [19,20,21,22,23]. The participant then completed digital versions of two standardized questionnaires—the Hospital Anxiety and Depression Scale (HADS) to assess anxiety and depression level, and the Neck Disability Index (NDI) to assess the degree of disability related to neck pain [24,25]. 

The HADS consists of 14 items, 7 corresponding to an anxiety subscale and 7 to a depression subscale, and asks the participant to consider the past seven days when choosing responses. Each item has four Likert-type response options scored from 0 to 3 points each for a maximum total score of 42 points. The Spanish version has been validated and demonstrates reliability ranging from 0.85 to 0.91 [26,27].

The NDI evaluates the effect of neck pain on activities of daily living and social relationships. It consists of 10 items with 6 response options that are scored from 0 to 5 points each for a total possible score of 50. The classification of disability is based on the total score obtained, with higher number indicating greater disability. This questionnaire demonstrates high test–retest reliability with ICC = 0.88 [25].

Measurement of cervical spine AROM was performed using the CROM system (Performance Attainment Associates, Roseville, MN, USA). The device consists of a plastic structure that is attached to the subject’s head as if it were a pair of glasses, with Velcro straps to secure it in place. Attached to this structure are three disc-shaped dials; two are inclinometers for measurements in the sagittal and frontal planes, and the third is a compass for measuring movements in the transverse plane [10,28].

**Figure 1:**
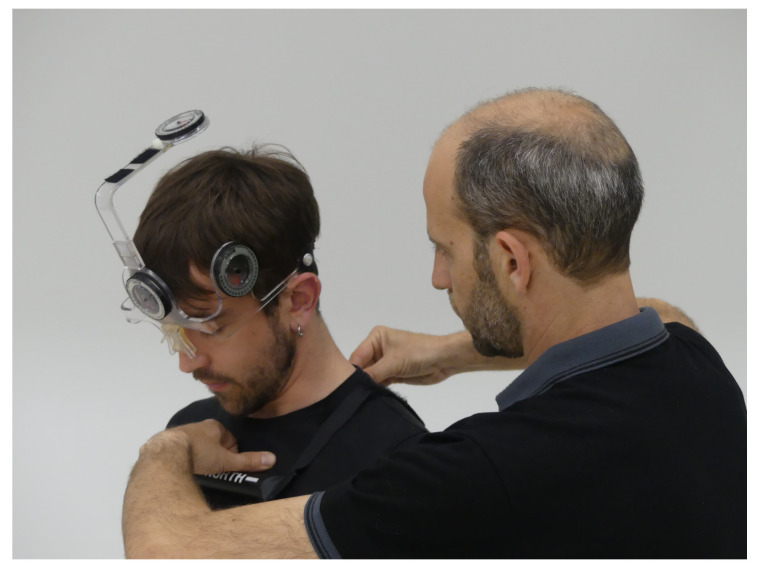
Position for measuring GCS flexion.

**Figure 2:**
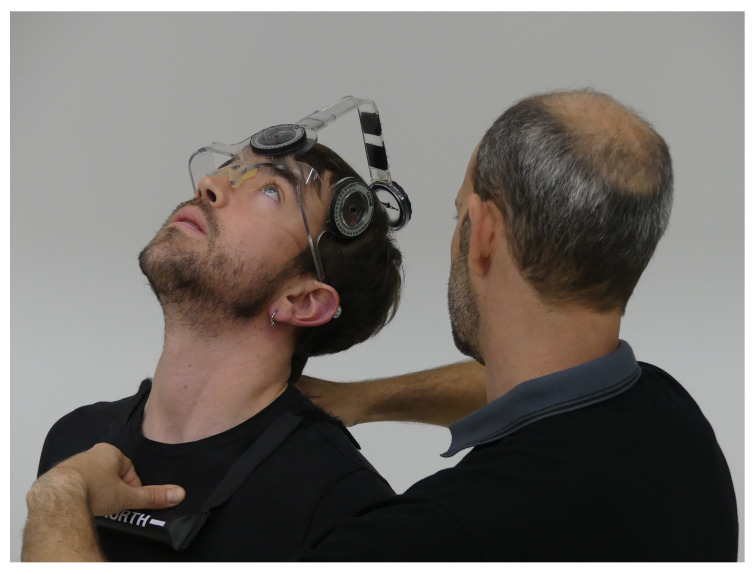
Position for measuring GCS extension.

In preparation for GCS AROM measurements, the participant was asked to sit in a chair with feet flat on the floor and knees flexed to 90°, such that their sacrum and thoracic spine were fully supported by the backrest. Prior to the start of the evaluations, the CROM system was adjusted to the participant’s head and the participant performed a warm-up consisting of three active movements in each direction of the cervical cardinal plane motions [10,18]. For all GCS AROM measurements, the evaluator was positioned at the left side of the participant to provide manual stabilization of the participant’s thoracic spine while reading the sagittal plane CROM dial. The participant was first instructed to perform cervical flexion AROM to the maximum symptom-free range and to stop at the end of the movement to facilitate inclinometer reading (Figure 1). The motion was performed three times and the mean of the three readings was used for statistical analysis. The participant was then instructed to perform cervical extension AROM in a similar manner, with the mean of three readings used for analysis (Figure 2). The test–retest reliability of this procedure in previous studies was 0.89 [18].

**Figure 3:**
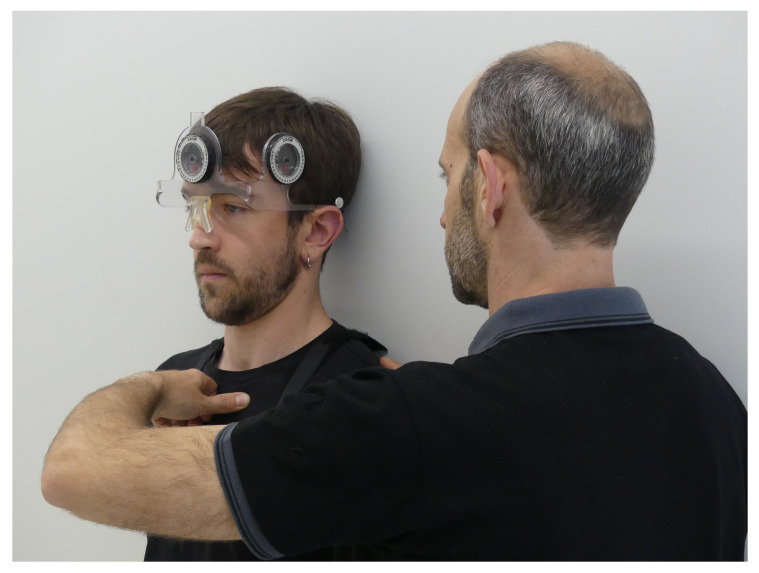
Procedure for measuring UC flexion.

**Figure 4:**
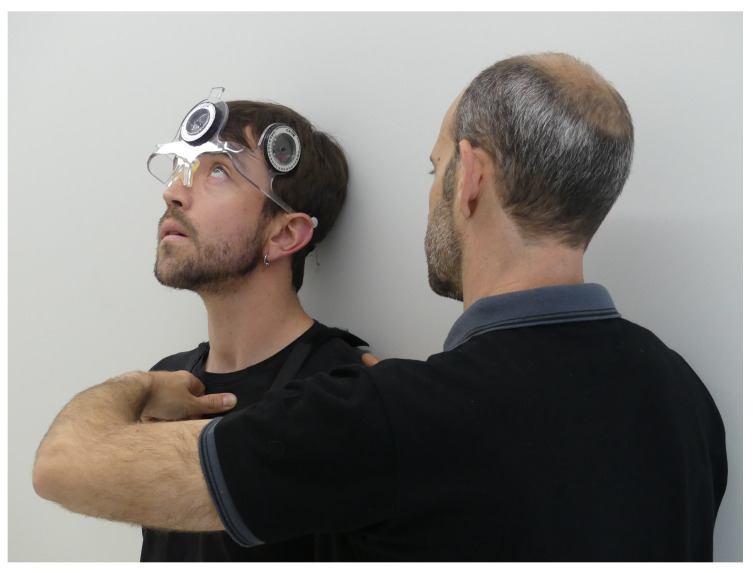
Procedure for measuring UC extension.

Sagittal plane UCS AROM was performed according to the methodology described by Dhimitri et al. [13]. The participant was positioned standing with their back toward a wall with the pelvis, thoracic spine, and head in contact with the wall [13,29]. The evaluator was positioned at the left side of the participant to provide manual stabilization of the participant’s sternum while reading the sagittal plane CROM dial. The participant was first instructed to perform UCS flexion, without losing head contact with the wall, to the maximum symptom-free range and maintain the final position to allow recording of the measurement (Figure 3). The procedure was performed three times and the mean was used for statistical analysis. The participant was then instructed to perform UCS extension in a similar manner, with the mean of three readings used for analysis (Figure 4). The intra-rater and inter-rater reliability of this procedure have been reported previously as 0.81 and 0.95, respectively [13].

### 2.3. Data Analysis

Descriptive statistics were utilized, including frequencies, percentages, and measures of central tendency. Correlation analysis was performed using Spearman’s rho. Linear regression models were developed to understand the relationship of age and sex with each AROM measurement. Data were analyzed using IBM SPSS Statistics version 20.0 (IBM Corp., Armonk, NY, USA) with a significance level set at *p* < 0.05.

## 3. Results

### 3.1. Participants 

**Scheme 1:**
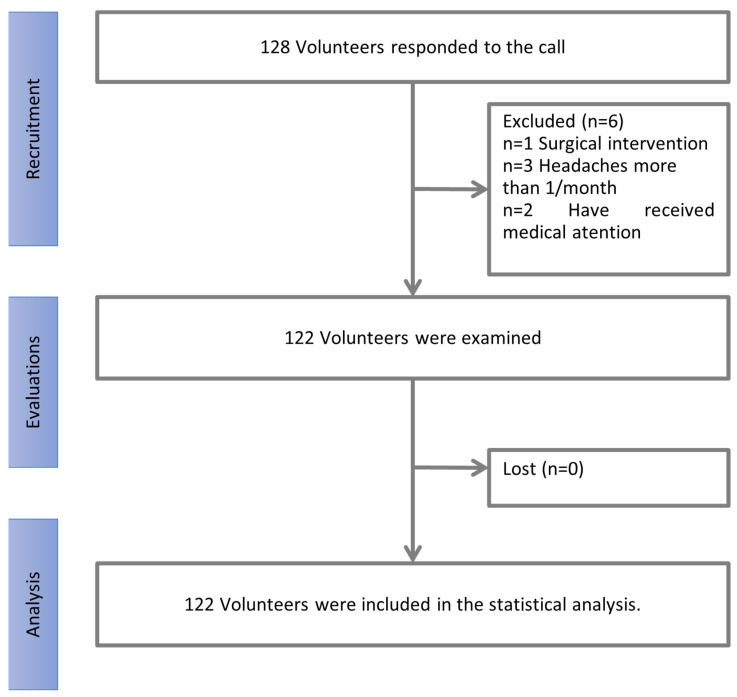
CONSORT flowchart of participants.

A total of 128 adults volunteered to participate in the study, 122 of which met the inclusion criteria and gave consent to participate. The reasons for exclusion were as follows: history of cervical spine surgery (1 participant), headaches more than once per month (3 participants), and having received medical care for a cervical complaint in the last year (2 participants). The CONSORT flowchart (Scheme 1) illustrates this process and reasons for exclusion.

Participant characteristics are summarized in Table 1. Sixty-one men and 61 women with a mean age of 45.16 (±16.76) years participated in the study, with 10 to 11 participants of each gender in each of the six age groups. Forty-eight (39.3%) participants reported having neck pain in the last year, 12 (9.8%) reported neck pain on the date of evaluation, and the mean NDI score was 1.80 (±2.65) indicating no associated disability.

### 3.2. Cervical AROM

Results of GCS and UCS flexion and extension AROM for the study sample are summarized in Table 2. The mean GCS AROM values were 39.94° (9.84) for flexion and 60.49° (10.28) for extension. The mean UCS AROM values were 8.74° (3.16) for flexion and 24.74° (6.86) for extension.

### 3.3. Linear Relationships between Cervical AROM, Age, and Sex

Active ROM stratified by age is described in Table 3, and Table 4 contains the results of linear regression models for each variable with age group as the only predictor and with adjustment for the other variables that had significance in the modeling process. The linear regression models indicate a significant decrease in AROM with increasing age for all motions except UCS flexion. Mean GCS flexion AROM decreased at a rate of 2.01° (*p* < 0.001) per decade of increasing age, beginning at 46.75° (9.96) in the 18–25 years age group and decreasing to 35.2° (9.51) in the 66–75 years age group. Mean GCS extension AROM also decreased with increasing age at a rate of 3.33° (*p* < 0.001) per decade, from 67.68° (7.53) in the 18–25 years age group to 51.1° (8.37) in the 66–75 years age group. Mean UCS extension AROM decreased at a rate of 1.87° (*p* < 0.001) per decade, from 28.3° (3.91) in the youngest age group to 19.05° (5.78) in the oldest age group.

Active ROM stratified by sex is described in Table 5, and Table 6 contains the results of linear regression models developed for each variable by sex. Linear regression models with significance indicated greater AROM in females for extension and total sagittal plane motion in the cervical spine as a whole and in the upper cervical region. Mean GCS and UCS flexion AROM did not demonstrate a statistically significant difference between sexes; however, there was a trend toward significance in GCS flexion AROM with men demonstrating 3.27° (*p* = 0.05) less motion than women. Mean GCS and UCS extension AROM each demonstrated a statistically significant difference between sexes, with linear regression coefficients of −6.52° (*p* < 0.001) for GCS extension and −2.43° (*p* = 0.023) for UCS extension, indicating decreased AROM in men compared with women for both variables.

### 3.4. Correlation between GCS and UCS AROM

Table 7 contains the results of the correlation analysis between GCS flexion and extension AROM variables and UCS flexion and extension AROM variables. The analysis indicates statistically significant correlations ranging from very weak, such as those found between UCS flexion and the total arc of sagittal plane GCS AROM (0.256), to strong, such as those found between the total arc of GCS AROM in the sagittal plane and both GCS flexion and GCS extension AROM (0.779, 0.838).

## 4. Discussion

The purposes of this study were to describe sagittal plane GCS AROM and UCS AROM in adults without cervical pathology, stratified by age and sex, and to compare GCS AROM with UCS AROM. The statistically significant findings include a decrease in AROM with increasing age for all variables except UCS flexion, males having decreased AROM compared with females for all variables except GCS flexion and UCS flexion, and correlations of varying strength between individual cervical AROM variables.

The mean values for global cervical flexion and extension AROM obtained in the current study were lower than those reported in the meta-analysis by Thoomes-de Graaf et al. [9]. The difference in cervical flexion AROM between the studies was approximately 15° in the younger age groups and 5° in the older age groups. Differences in cervical extension AROM were smaller, at 7° difference in the younger age groups and 3° in the older groups. This pattern may be due to the greater number of studies of younger participants included in the meta-analysis, which introduces greater variability in measurement methods in the pooled data for the younger age groups. Of the studies included in the meta-analysis, only one included individuals older than age 70, and these results are closer to those of the current study. 

With respect to age, the meta-analysis by Pan et al. reported trends similar to those of the current study, including a decrease in cervical flexion AROM between the 20–30 years and 40–50 years age groups and a decrease in cervical extension AROM between the 20–30 years and 50–60 years age groups; however, the methods of statistical analysis differed between the studies [8]. With respect to sex, the results of the current study are also similar to those of Pan et al., who reported that total sagittal plane AROM (flexion and extension combined) was greater in women than in men.

The meta-analysis by Chen et al. includes results for cervical flexion and extension AROM measured by inclinometry, a system similar to the CROM, with values of 61° and 77°, respectively (20–50 years) [7]. The difference in AROM values compared with our results, cervical flexion 39.94° (9.84) and cervical extension 60.49° (10.28) (18–75 years), may reflect methodological differences. In addition, differences in the age of participants could have influenced this difference in results. Some studies included in the meta-analysis did not describe any methodology used to stabilize the thoracic spine, while others only asked the subjects to keep their thoracic region stable [30,31]. Additionally, one study indicated that the participant’s arms remained supported on armrests, which could reduce tension in the scapulo-cervical musculature and allow increased cervical AROM [31]. With respect to age and sex, narrative analysis by Chen suggested a decrease in AROM of 4° per decade for all movements, and greater ROM in women compared to men [7]. 

The inclusion of subjects having subclinical neck pain, although without significant disability, may have contributed to lower ranges of motion in the current study. Other sources of disparity in the results may be related to characteristics of the study samples. 

Two prior studies are available for comparison of sagittal plane UCS AROM in participants without pathology, both of which employed methods to limit lower cervical spine motion [13,14]. These studies report UCS flexion AROM between 4° and 15.03° and UCS extension AROM between 11.2° and 23.32° [13,14]. The results of the current study, UCS flexion 8.74° (3.16) and UCS extension 24.74° (6.86), are most similar to those obtained by Amiri et al. The methodology used in the current study, however, was very similar to that of Dhimitri et al. in which the participant’s skull maintained contact with the wall at all times, without palpation or manual stabilization of the C2 vertebra [13]. Amiri et al. utilized only manual stabilization of C2 without skull contact on the wall [14]. An investigation comparing UCS AROM values obtained with these two methods individually as well as combined may inform future studies and clinical best practices for measuring AROM isolated to the upper cervical region.

Loss of ROM with age is expected due to age-related changes in joints and ligament orientation and articular surface areas [32]. Results showed a loss of GCS AROM that could be related to the effects of aging such as spondylosis, showing the effect of dehydration and shrinking of cervical intervertebral discs and osteoarthritis development [33,34]. However, results in UCS showed a loss of AROM for extension but not for flexion. This fact is consistent with previous studies that consider that age-related changes in UCS remain unclear [5]. The difference in loss of ROM between the two regions could be due to the lower cervical spine being more affected by spondylosis and loss of thickness of intervertebral discs.

The sagittal plane cervical spine AROM findings in the current study are consistent with the results of some previous publications and differ in comparison with others. Considering the differences in methodology across the available studies, it seems necessary to establish a standardized method for measurement and reporting. One possible consideration for standardizing the start position is the alignment of the Frankfurt plane, an imaginary line from the upper edge of the external auditory canal to the base of the orbit of the eye, with the horizontal plane [4]. Another methodological aspect that requires standardization is the stabilization of the lower cervical spine to consistently identify the cessation of UCS movement, which could be addressed by adding manual stabilization of C2 during UCS flexion and extension AROM. 

No studies were located that reported sagittal plane UCS AROM with a sample size similar to ours, and none of the previously published studies have analyzed the influence of sex and age on UCS flexion and extension AROM. Therefore, the results of this study may provide a reference for future research and be useful in the clinical setting when evaluating patients with cervical spine pathology. 

### Limitations

While the cross-sectional design of this study identified relationships among the variables studied, it is not possible to establish causality. The use of convenience sampling among members of a university community may present a selection bias related to socioeconomic and employment characteristics, and extrapolation of the results is limited to populations meeting the inclusion and exclusion criteria. These aspects limit the use of these values as normative cut-off values between asymptomatic/subclinical and symptomatic populations. The age strata used in this study are different than those used in other publications, which creates challenges for comparison and inclusion of the results in future meta-analyses. The fact that a single examiner, who was not blinded to the measurements, performed all the measurement procedures of the study limits the external validity of the results. Future studies aimed at understanding the functional characteristics of the cervical spine should include multiple evaluators. Likewise, it is recommended that the evaluator be blinded to the measurements through the use of digital tools.

## 5. Conclusions

In summary, this study contributes reference values for GCS and UCS flexion, extension, and total sagittal plane AROM in adult men and women without cervical pathology across several decades of the lifespan. Women demonstrated statistically greater motion than men in extension and total sagittal plane AROM in both the upper and lower cervical regions and demonstrated a trend toward greater motion in GCS flexion. A pattern of a statistically significant decrease in AROM with increasing age was present at varying magnitudes in all variables except UCS flexion. Clinicians should consider these age- and sex-related patterns when examining the AROM of patients seeking care for cervical spine region pain or pathology, when identifying impairments, and when setting treatment goals related to range of motion. Clinicians should also recognize that variability exists in the literature around this topic, some of which may be related to differences in measurement methods. Accordingly, AROM measurement in clinical practice should aim to replicate the measurement methods used to derive the reference AROM values to which the motions will be compared in order to increase the accuracy in identifying impaired motion when present.

## Figures and Tables

**Table 1:** Descriptive data for independent variables in the whole sample as minimum, maximum, mean, and standard deviation.

n = 122	Minimum	Maximum	Mean	Standard Deviation
Age	18	75	45.16	16.76
Hours of use of data display screens/day	0.5	14	6.61	3.53
Sitting hours/day	1	15	7.49	3.31
Hours of physical activity/week	0	40	7.26	6.84
Days of physical activity/week	0	7	4.17	2.27
HADS tot (42)	0	25	6.99	4.68
NDI (50)	0	13	1.80	2.65
	Frequency		Percentage	
Men	61		50	
Women	61		50	
Visual handicap	83		68	
Dental splint	29		23.8	
Drug use	34		27.9	
Neck pain during last year	48		39.3	
Current neck pain	12		9.8	

**Table 2:** Sagittal plane descriptive statistics of the whole sample.

	N	Min.	Max.	Mean	SD
ROM CS Flexion	122	12.67	69.33	39.94	9.84
ROM CS Extension	122	32.67	83.33	60.49	10.28
ROM CS Sagittal	122	59.33	138.67	100.43	16.30
ROM UCS Flexion	122	2.67	18.00	8.74	3.16
ROM UCS Extension	122	6.00	45.67	24.74	6.86
ROM UCS Sagittal	122	14.00	52.67	33.48	7.78

**Table 3:** Results of variables in the sagittal plane by age group.

	18–25	26–35	36–45	46–55	56–65	66–75
	Mean (SD)	Mean (SD)	Mean (SD)	Mean (SD)	Mean (SD)	Mean (SD)
ROM CS Flex	46.75 (9.96)	39.51 (11.83)	41.75 (10.47)	41.08 (4.66)	35.3 (6.85)	35.2 (9.51)
ROM CS Ext	67.68 (7.53)	66.44 (8.56)	62.58 (9.99)	58.57 (9.46)	56.35 (7.91)	51.1 (8.37)
ROM CS Sagittal	114.43 (13.45)	105.95 (15.78)	104.33 (17.44)	99.65 (11.93)	91.65 (9.87)	86.3 (12.2)
ROM UCS Flex	9.42 (2.08)	8.7 (3.02)	8.3 (3.79)	9.08 (3.18)	8.98 (3.59)	7.95 (3.17)
ROME UCS Ext	28.3 (3.91)	27.65 (5.76)	27.23 (5.1)	25.02 (7.95)	21.03 (6.73)	19.05 (5.78)
ROM UCS Sagittal	37.72 (4.7)	36.35 (5.79)	35.53 (5.66)	34.1 (9.57)	30.02 (7.21)	27 (7.61)

**Table 4:** Linear regression models of variables in the sagittal plane by age group.

	Unadjusted Difference between Groups (95% CI)		Adjusted Difference between Groups (95% CI)		
	Value	*p*	Value	*p*	Adjusted by
ROM CS Flex	−2.01 (−2.99, −1.04)	0.000	−2.01 (−2.99, −1.04)	0.000	No other variable showed significance in the model
ROM CS Ext	−3.35 (−4.26, −2.45)	0.000	−3.33 (−4.17, −2.5)	0.000	Sex
ROM CS Sagittal	−5.37 (−6.79, −3.94)	0.000	−5.41 (−6.73, −4.09)	0.000	Sex, NDI
ROM UCS Flex	−0.16 (−0.49, 0.17)	0.340	−0.3 (−0.64, 0.04)	0.086	h/week PA
ROM UCS Ext	−1.95 (−2.58, −1.32)	0.000	−1.87 (−2.54, −1.21)	0.000	Sex, discharge splint, medication intake, NDI
ROM UCS Sagittal	−2.11 (−2.84, −1.38)	0.000	−2.38 (−3.05, −1.7)	0.000	Sex, discharge splint, NDI

**Table 5:** Results by sexes of the variables in the sagittal plane.

	Men	Women
	Mean (SD)	Mean (SD)
ROM CS Flex	38.27 (8.33)	41.61 (10.96)
ROM CS Ext	57.17 (9.92)	63.8 (9.61)
ROM CS Global	95.44 (14.05)	105.41 (16.96)
ROM UCS Flex	8.45 (2.83)	9.03 (3.45)
ROM UCS Ext	23.27 (7.48)	26.21 (5.87)
ROM UCS Sagittal	31.72 (8.21)	35.24 (6.96)

**Table 6:** Linear regression models of variables in the sagittal plane by sex.

	Unadjusted Difference between Men and Women (95% CI)		Adjusted Difference between Men and Women (95% CI)		
	Value	*p*	Value	*p*	Adjusted by
ROM CS Flex	−3.34 (−6.83, 0.15)	0.061	−3.27 (−6.55, 0.01)	0.050	Age group
ROM CS Ext	−6.63 (−10.13, −3.13)	0.000	−6.52 (−9.37, −3.67)	0.000	Age group
ROM CS Sagittal	−9.97 (−15.55, −4.38)	0.001	−10.53 (−15.07, −5.99)	0.000	Age group, NDI
ROM UCS Flex	−0.58 (−1.72, 0.55)	0.308	−0.76 (−1.88, 0.36)	0.183	h/week AF
ROM UCS Ext	−2.93 (−5.34, −0.52)	0.017	−2.43 (−4.51, −0.35)	0.023	Age group, discharge splint, medication intake, NDI
ROM UCS Sagittal	−3.52 (−6.25, −0.79)	0.012	−3 (−5.36, −0.65)	0.013	Age group, discharge splint, NDI

**Table 7:** Correlations between variables in the sagittal plane.

		ROM CS Flex	ROM CS Ext	ROM CS Sagittal	ROM UCS Flex	ROM UCS Ext	ROM UCS Sagittal
ROM CS Flex	Spearman’s rho	-	0.340 **	0.779 **	0.331 **	0.380 **	0.481 **
	*p*	-	0.000	0.000	0.000	0.000	0.000
ROM CS Ext	Spearman’s rho	0.340 **	-	0.838 **	0.104	0.507 **	0.502 **
	*p*	0.000	-	0.000	0.256	0.000	0.000
ROM CS Sagittal	Spearman’s rho	0.779 **	0.838 **	-	0.256 **	0.561 **	0.613 **
	*p*	0.000	0.000	-	0.004	0.000	0.000
ROM UCS Flex	Spearman’s rho	0.331 **	0.104	0.256 **	-	0.124	0.530 **
	*p*	0.000	0.256	0.004	-	0.172	0.000
ROM UCS Ext	Spearman’s rho	0.380 **	0.507 **	0.561 **	0.124	-	0.880 **
	*p*	0.000	0.000	0.000	0.172	-	0.000
ROM UCS Sagittal	Spearman’s rho	0.481 **	0.502 **	0.613 **	0.530 **	0.880 **	-
	*p*	0.000	0.000	0.000	0.000	0.000	-

**. Correlation is significant at the 0.01 level (bilateral).

## Data Availability

The data supporting reported results are available upon reasonable request to the communication authors.
